# Neurophysiological Correlates of Target-Related Visual Attention Processes as a Function of Reward in Connection with Schizotypy

**DOI:** 10.1007/s10548-026-01260-0

**Published:** 2026-10-10

**Authors:** Nils Hübner, Denise Lockhofen, Ranjan Debnath, Christoph Mulert

**Affiliations:** Centre of Psychiatry, Justus-Liebig University Giessen, Klinikstrasse 36, 35392 Giessen, Hessen, Germany

**Keywords:** Schizotypy, EEG, Reward, Visual attention, N2pc

## Abstract

In everyday life, we constantly select and perceive relevant information while suppressing irrelevant sensory input. This selection process is governed by the interaction between bottom-up and top-down attention mechanisms. Recent studies also highlighted the influence of reward on attention, with monetary rewards shown to enhance task performance and modulate stimulus salience. Schizotypy, conceptualized as a continuum ranging from healthy states to schizophrenia symptoms, provides a framework for the investigation of these mechanisms in non-clinical populations. This study investigated the behavioral and neurophysiological correlates of visual attention and reward processing, and their relationship with schizotypy traits. Thirty-six healthy participants were divided into two groups which performed a visual attention task with salient distractors and rewarded (target reward group, TR) or not rewarded (no reward group, NR) target stimuli while EEG was recorded. Additionally, schizotypy was assessed using the Schizotypal Personality Questionnaire (SPQ). The results showed that salient, but task-irrelevant distractors increased response times compared to target-only trials irrespective of reward. On the neurophysiological level, distractors decreased target prioritization in the NR group. Furthermore, participants with higher scores on the negative schizotypy dimension showed stronger target prioritization, especially in the absence of salient distractors. These findings suggest that monetary rewards can modulate attentional control mechanisms and that negative schizotypy traits are linked to altered attentional prioritization.

**Supplementary Information:**

The online version contains supplementary material available at https://doi.org/10.1007/s10548-026-01260-0.

## Theory

In everyday life, we are surrounded by countless stimuli (sounds, odors, noises, visual stimuli) and are constantly challenged to select and perceive those that are most relevant to us (Desimone and Duncan 1995; Posner 1980). Thus, we must focus on relevant information while simultaneously suppressing irrelevant sensory input (Bellebaum et al. 2012; Lockhofen et al. 2021). This selection process is commonly explained in terms of bottom-up and top-down mechanisms (Corbetta and Shulman 2002). Bottom-up processes are thought to be driven implicitly by physical salience, such as color or orientation (Corbetta and Shulman 2002). A salient stimulus is easily detectable in the environment and can be processed rapidly and with high priority (Duncan and Humphreys 1989). In contrast, top-down processes are under voluntary control, guided by goals, intentions, and prior experience. However, this strict dichotomy might be overly simplistic. In real-world situations, top-down and bottom-up mechanisms often interact to guide attention. For example, when searching for someone in a crowd, objects of a certain color will stand out if the person is expected to be dressed in that color. This process helps us to focus on specific properties of our environment and modulate stimulus salience accordingly (Corbetta and Shulman 2002). Although attention has long been described in terms of this top-down versus bottom-up dichotomy, more recent studies highlight the importance of additional factors, such as reward (Awh et al. 2012). Cognitive tasks are performed more efficiently when subjects are rewarded (Rowe et al. 2008; Walsh et al. 2019), and even the expectation of reward alone can have a positive influence on task performance (Engelmann et al. 2009). Monetary rewards, in particular, have been shown to bias attention toward task-relevant stimuli (Engelmann et al. 2009; Libera and Chelazzi 2006).

Certain psychiatric conditions, such as paranoid schizophrenia, are characterized by alterations in visual attention and reward processing compared to healthy individuals. The main symptoms of schizophrenia include delusions, hallucinations, disorganized or catatonic behavior, and negative symptoms (Dilling et al. 2015). Typically, schizophrenia begins with a prodromal phase in younger adulthood (Marshall et al. 2014; McCutcheon et al. 2020), which occurs before the full onset of psychotic symptoms, and is characterized by gradual changes in behavior and cognition. Symptoms are often nonspecific and can resemble those of other mental health conditions, making diagnosis challenging. Therefore, a key objective of current schizophrenia research is to identify potential predisposing factors that contribute to increased vulnerability. Several studies have examined altered visual perception and stimulus processing in patients with schizophrenia. For example, patients with schizophrenia may exhibit disturbed motion perception (Stuve et al. 1997), altered or even dysfunctional organization of visual perception (Uhlhaas et al. 2005), impaired color perception (Shuwairi et al. 2002), and slower reactions to visual stimuli (Arnado and Olié 2006). Patients might also show deficits in reward anticipation and reward learning (Moran et al. 2019). Furthermore, the reward system in schizophrenic patients can be disrupted, particularly in the presence of negative symptoms such as depressive symptoms and anhedonia (Der-Avakian and Markou 2012). Another crucial difference between patients with schizophrenia and healthy subjects is their limited ability to distinguish between relevant and irrelevant information adequately. For example, they may incorrectly attribute motivational salience to neutral events or stimuli – a phenomenon known as aberrant salience (Kapur 2003). Several studies have found that schizophrenic patients experience difficulties in suppressing interfering information or distractors (Barch and Ceaser 2012; Henik and Salo 2004; Laurenson et al. 2015; Popov et al. 2019) and tend to hyper focus on irrelevant information (Luck et al. 2019; Hahn et al. 2022).

However, deficits in attention and reward processing are not only observed in patients diagnosed with schizophrenia but can also be found in individuals with elevated levels of schizotypy (Ettinger et al. 2015). Schizotypy refers to a continuum of experiences ranging from normal states to extreme symptoms associated with schizophrenia spectrum disorders (Raine 1991). These symptoms may include strange or bizarre behavior, unusual language, magical thinking, unusual perceptual experiences, and social anhedonia (Nelson et al. 2013). As with schizophrenia, schizotypy comprises three different dimensions: positive, negative, and disorganized (Wuthrich and Bates 2006). Several studies have shown that schizotypy is a valid measure for indicating psychosis proneness (Barrantes-Vidal et al. 2015; Debbané et al., 2015; Grant et al. 2018; Kwapil and Barrantes-Vidal 2015) and is a widely used dimensional marker of psychosis risk (Taylor et al. 2020). Especially the negative dimension is assumed to be an important risk factor for schizophrenia (Fanous et al. 2001) and has been shown to be associated with the occurrence of psychosis (Demjaha et al. 2012; Pelizza et al. 2020; Piskulic et al. 2012). Therefore, studying schizotypal traits in healthy individuals may provide further insights into the pathophysiology and etiology of schizophrenia. As mentioned above, schizotypy and schizophrenia appear to share not only symptomatic features but also deficits in perceptual, cognitive, and motor processing (Ettinger et al. 2015). Abnormalities in visual information processing (Teufel et al. 2015) and attention (Ducato et al. 2008) can be found in patients with schizophrenia as well as in individuals with schizotypal traits. For example, it has been demonstrated that participants with high schizotypy scores show low-level vision deficits (Harper et al., 2020; Llapashtica et al. 2025) that are observed in schizophrenia patients (Butler et al. 2008). Furthermore, patients with schizophrenia and participants with high schizotypy scores show abnormalities in attentional capture (Ducato et al. 2008) and demonstrate increased distractibility (Marsh et al., 2017; Grillon et al. 1990; Spring et al. 1989). Thus, assessing schizotypy scores alongside behavioral and neurophysiological correlates may provide insights into predisposing factors for psychotic illness in healthy populations. Some studies even suggest that neurophysiological measures associated with visual perception tasks may serve as potential biomarkers (Fuggetta et al. 2014). For example, early visual processing deficits found in participants with elevated schizotypy scores were associated with reduced P1 components during electroencephalography (EEG; Koychev et al. 2010). At the same time, individuals with high schizotypy scores have been shown to exhibit increases in EEG parameters associated with the processing of target stimuli (Fuggetta et al. 2015). Specifically, the late phase of the N2-posterior-contralateral (N2pc) evoked by target stimuli showed greater peak latency and amplitude in participants with high schizotypy, indicating abnormalities in endogenous control processes involved in disengaging attention from uninformative cues.

The influence of rewards on visual information processing abnormalities is still not completely understood. Since visual attention and reward processing are closely intertwined and schizophrenic patients show deficits in both domains, it is important to disentangle their independent effects at both behavioral and neurophysiological levels. Visual search tasks provide a well-suited paradigm to investigate the neurophysiological correlates of target processing and their modulation by reward. Numerous studies have shown that participants perform attention tasks faster or more accurately if they expect monetary rewards (Chelazzi et al. 2013; Shomstein and Johnson 2013; Yantis et al. 2012), likely due to an increased preparation (also known as priming) for task-related processes (Kiss et al. 2009). However, task performance can be impaired by introducing distractors in visual search tasks, providing a means to examine how irrelevant stimuli interfere with target processing (Anderson et al. 2013; Bourgeois et al. 2017; Chelazzi et al. 2013; Hickey et al. 2010; Lockhofen et al. 2021; Rusz et al. 2020; Watson et al. 2019a, b). Thus, visual search tasks can be designed to both facilitate and disrupt performance in order to examine the effects of reward and distractors on attentional processing.

In the present study, we employed a variant of the Additional Singleton Task (AST) with rewarded or non-rewarded targets and salient distractors (see Feldmann-Wüstefeld et al. 2016; Lockhofen et al. 2021, 2025). Participants were instructed to correctly identify a target within a heterogeneous search matrix while ignoring a colored distractor. In a previous study with a similar design, we found that monetary rewards had a positive effect on target processing, as demonstrated by faster response times for high-reward targets compared to low-reward targets (Lockhofen et al. 2021). This effect was only observed when targets, not distractors, were rewarded. To further investigate this effect, the current study compared a group with rewarded targets (TR) with a no-reward group (NR). A key advantage of the AST is that it allows the investigation of neurophysiological processes using EEG (Feldmann-Wüstefeld et al. 2016). This study analyzed the N2pc event-related potential to examine attention-related neural processes. The N2pc consists of several subcomponents: target negativity (NT), distractor negativity (ND), and distractor positivity (PD). ND reflects attentional capture by and PD reflects active suppression of the distractor, whereas NT indicates target prioritization. By using a systematic lateralization technique (Hickey et al. 2009; Woodman and Luck 2003), it is possible to separate these subcomponents of the N2pc. For this study, the NT component, reflecting target prioritization, was of particular interest.

The aim of the present study was to investigate the behavioral and neurophysiological correlates of visual attention and reward processing, and their relationship with schizotypy traits in healthy participants. We hypothesized that rewarding targets would facilitate target processing, resulting in faster response times and increased NT amplitudes, compared with the No-Reward group. On the other hand, we expected that the presence of distractors would impair target processing, leading to slower response times and decreased NT amplitudes compared to target-only trials. Schizotypy was measured using the Schizotypal Personality Questionnaire (SPQ). We expected that higher schizotypy scores would be associated with increased distractibility and thus with slower response times and reduced NT amplitudes.

## Method

### Ethics Statement

The study was approved by the Institutional Review Board of the Justus Liebig University Giessen and carried out in accordance with the Declaration of Helsinki. Written informed consent was obtained from all participants.

### Participants

The final sample consisted of 36 healthy participants, evenly assigned to a target reward (TR) group (*n* = 18) and a no reward (NR) group (*n* = 18), after excluding four individuals due to technical issues. A post hoc power analysis using G*Power indicated that the study had high achieved power (1 − β = 1.00) for detecting the Distraction × Group interaction, based on the observed effect size (ηp² = 0.31; f = 0.67), α = 0.05, and the final sample size (*N* = 36). The mean age was 24.6 years (SD = 4.41, female/male: 8/10) in the TR group and 25.5 years (SD = 4.48, female/male: 7/11) in the NR group. The two groups did not significantly differ in age (t(34) = 0.576, *p* = .568) or sex distribution (*χ*^2^(36) = 0.114, *p* = .735). All participants were right-handed, had normal or corrected-to-normal vision, and had no color blindness as verified using the Snellen vision test and the Ishihara Test for Color Deficiency. Individuals with a history of psychiatric or neurological disorders were excluded. Participants were recruited via email announcements at the Justus Liebig University Giessen. Efforts were made to ensure a diverse educational background for broader representativeness. Eligibility was confirmed through a screening phone call. Participants received either €8 per hour or test subject hours, a solidarity-based, university-wide system to ensure the availability of participants, in addition to the reward from the Task (approximately 20€).

### Experimental Design and Paradigm

The Additional Singleton Task (AST; Theeuwes 1991) is a visual search paradigm designed to investigate the interplay between bottom-up and top-down attentional mechanisms. The present study employed a modified version of the AST to include reward manipulation (Feldmann-Wüstefeld et al. 2016). In this modified version of the AST, participants were required to identify and respond to a target line with a unique orientation (“/” or “\”) presented among a heterogeneous array of horizontal and vertical lines. In 70% of the trials, a color singleton distractor (red or blue) was also presented to induce attentional interference, whereas the remaining 30% were target-only (TO) trials.

Participants were randomly assigned to one of two groups: a Target Reward (TR) group, in which correct responses to a specific target orientation were rewarded with points (displayed as “+10”), and a No Reward (NR) group, which received only accuracy feedback (“0” or “+1”). Participants were not informed about the reward contingency beforehand, but were told that the accumulated points would be converted into a monetary bonus at the end of the experiment (TR group). The association between target orientation and reward had to be learned implicitly during the experiment. The specific target orientation associated with reward (“/” or “\”) was counterbalanced across participants in the TR group. One thousand points corresponded to €4.19, with a maximum possible payout of 5808 points (€24.39). All participants in the TR group earned over €20, and NR participants received a fixed amount of €22 to ensure fairness.

**Fig. 1:**
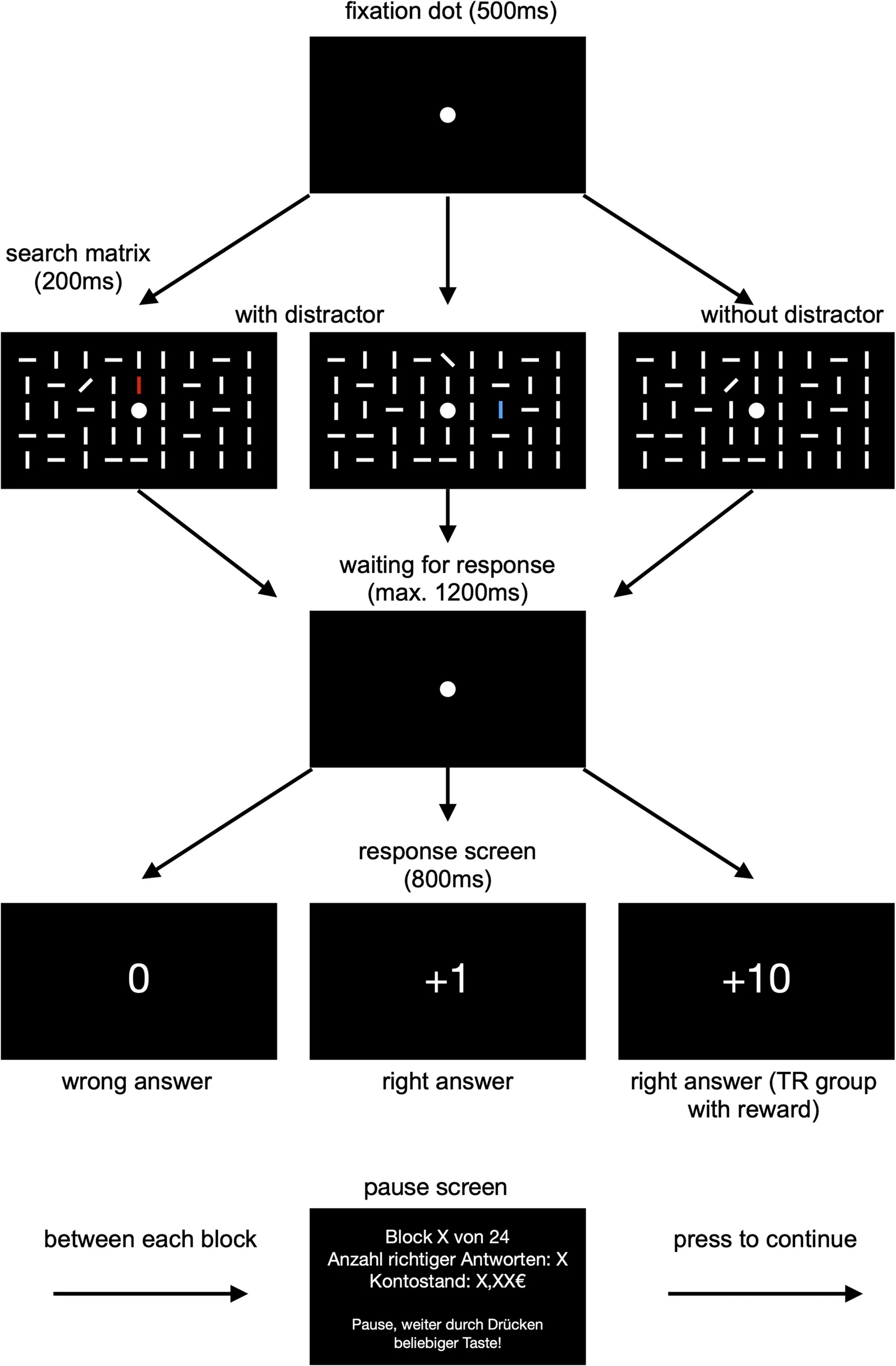
Illustration of a single trial in the AST. The feedback of “+10” was presented only in the TR group following a correct response in the rewarded condition. During the pause screen, participants were shown the current block number, the number of correct responses, and accumulated monetary earnings. Figure adapted from Lockhofen et al. (2021)

The task consisted of 1056 trials, divided into 24 blocks of 44 trials each. Each block included 32 distractor-trial combinations and 12 target-only trial types. Each combination was presented once per block. Each trial began with a fixation cross (500 ms), followed by a stimulus array (200 ms) and a response window of up to 1200 ms. Feedback was provided immediately after the response for 800 ms. Participants could take self-paced breaks between blocks, during which their cumulative points and earnings were displayed (Fig. 1).

### Procedure

Data collection took place at the Centre for Psychiatry in Giessen. Upon arrival, participants were provided the informed consent form, privacy policy, and experimental instructions. They were given sufficient time to read and sign these documents. A staff member was available to answer any questions. Once documentation was completed, the vision tests were conducted. After confirming eligibility, participants proceeded to the experiment room. Stimuli were displayed on a 23.8-inch LCD monitor (Asus VZ249HE-W) using *Presentation* software (Neurobehavioral Systems,^2023^). Participants were seated approximately 130 cm from the screen. The visual array consisted of a 27 × 17 matrix (visual angle: 20° × 13°) of white lines (RGB: 134,134,134) on a dark gray background (RGB: 60,60,60). Target lines were tilted 45°, and colored singleton distractors appeared in red (RGB: 232,34,34) or blue (RGB: 82,124,255). Target and distractor positions followed a fixed scheme across six predefined locations: two central (above/below fixation) and four laterals (left/right of fixation). On each trial, one item was presented centrally (above or below fixation) and the other laterally (left or right of fixation).

Before starting the experiment, participants completed a practice session; first at a slower pace, followed by a session at normal speed. Participants were informed that the money earned during the practice session would not be transferred to the actual experiment, which always started with an account balance of 0. The practice session allowed participants to familiarize themselves with the task and ensured proper functioning of the EEG. After addressing any remaining questions, the experiment commenced and lasted approximately 90 min. After the EEG session, participants completed a set of questionnaires. Finally, they received compensation via bank transfer or test subject hours. After the experiment, participants were asked whether they had noticed the colored distractor and the orientation–reward association. All participants recognized the distractor, and only two participants in the TR group failed to recognize the reward contingency.

### EEG-recording

EEG data were recorded using a 64-channel EEG system with Ag/AgCl electrodes mounted on elastic caps (ActiCaps, Brain Products, Munich, Germany). Data acquisition was performed with the Brain Vision Recorder software (Brain Products GmbH, 2023). The electrode layout followed a modified 10/20 system, which excluded electrodes FPz, F9, F10, CP3, CP4, P9, P10, and PO8. Additional electrodes were placed at PO9 and PO10. Eye movements were recorded using four electrooculography (EOG) channels, placed at the outer canthi of the right eye, infra- and supraorbitally. FCz served as the reference electrode, and AFz was used as the ground electrode. Head circumference was measured to select the appropriate cap size, and electrolyte gel was used to reduce impedance. Impedance was maintained below 5 kΩ for all electrodes. The preparation process took approximately 30 min.

### Questionnaires

Demographic data were collected using the Gießener Erhebungsbogen zu Krankengeschichte und psychosozialen Status (GEKS), a tool developed by the Centre for Psychiatry in Giessen. Handedness was assessed using the Edinburgh Handedness Inventory (Oldfield, 1971). Schizotypal traits were measured using the Schizotypal Personality Questionnaire (SPQ), which consists of 74 items answered with “yes” or “no”. This questionnaire was specifically designed to assess schizotypal experiences and validated for use in healthy populations (Kornmayer et al. 2015; Raine 1991). The mean SPQ total score in the sample was 10.2 (range: 1–34, SD = 8.73, Cronbach’s α = 0.92). In addition, we calculated three SPQ subscales: positive, negative, and disorganized. For the positive subscale, the mean was 4.42 (range: 0–15, SD = 4.72, Cronbach’s α = 0.85). The negative subscale had a mean of 3.14 (range: 0–14, SD = 3.19, Cronbach’s α = 0.81), and the disorganized subscale had a mean of 2.64 (range: 0–12, SD = 2.58, Cronbach’s α = 0.80).

### Data Analysis

Jamovi (The Jamovi Project, 2022) and R were used for statistical analyses. Behavioral and ERP data were analyzed using mixed-design ANCOVAs with Distraction (Distractor, Target-Only) as a within-subjects factor, Group (TR, NR) as a between-subjects factor, and mean-centered Age, Gender, and SPQ scores as covariates. Separate models were run for the SPQ Total score and its Disorganized, Positive, and Negative subscales. Only the SPQ Negative subscale showed in ERP a significant interaction with Distraction; however, results for the other models were computed and reported in the supplementary material for completeness.

All statistical tests were conducted at a significance level of α = 0.05 and all relevant statistical assumptions for mixed-design ANCOVA were evaluated and met.

#### Questionnaire Data

The evaluation of the subscales of the Schizotypal Personality Questionnaire (SPQ) was conducted in accordance with Kornmayer et al. (2015) and Raine et al. (1994). The positive dimension comprises four subscales: Ideas of reference, Magical thinking, Unusual perceptual experiences, and Paranoid ideation (Suspiciousness). The negative dimension comprises three subscales: Social anxiety, No close friends, and Constricted affect. The disorganized dimension comprises two subscales: Odd behavior and Odd speech.

#### Behavioral Data

The analysis of response times from the logfiles of the AST recordings was performed using DataWeasel (Ruprecht et al. 2017), a software developed at the Center for Psychiatry in Giessen. This software calculates response times for individual trials and subsequently groups them according to specific criteria. The response time was defined as the interval between releasing the hold key and pressing the response key. Trials with incorrect responses or responses time exceeding 1200 ms were excluded from the analysis. For each participant, the mean response time was calculated for each condition across trials. The primary focus of this study was to investigate the effects of Reward (TR vs. NR) and Distraction (distractor vs. target-only trials). Therefore, only trials suitable for the calculation of target negativity (i.e., targets presented at lateral positions) were included. This design allowed investigation of how reward and distractor influenced response times and whether these effects were modulated by schizotypal traits.

#### EEG Data Processing

The analysis of EEG data was performed using BrainVision Analyzer software (Brain Products GmbH, 2019). Preprocessing of raw EEG data was identical for all participants in both groups. Following preprocessing, the N2pc and its subcomponent, the Target-Negativity (NT), were calculated. After analysis in BrainVision Analyzer, individual participants’ data were exported in Jamovi and R for statistical analyses.

##### Preprocessing of EEG Data

The raw EEG data were imported into the BrainVision Analyzer workspace for preprocessing. Due to technical issues, electrodes *Fp1* and *Fz* were excluded for all participants and replaced via interpolation (*Fp1* = (AF7 + AF3)/2; *Fz* = (F1 + F2)/2, in µV). Subsequently, both channels were removed from the dataset, and the channel order was adjusted for clarity. A high-pass filter (0.5 Hz, order 4), a low-pass filter (15 Hz, order 8), and a notch filter (50 Hz, bandwidth 10, order 2) were applied to the continuous EEG data. Ocular artifacts were corrected using Independent Component Analysis (Fast-ICA-Restricted, 150 steps, convergence threshold 1E-07). The data were re-referenced to an average reference and automatically inspected for artifacts using the following thresholds: amplitude jumps > 50 µV/ms, amplitudes exceeding ± 200 µV, and minimum activity < 0.3 µV. A 200 ms window before and after each artifact was marked, with affected intervals comprising less than 1% of the data. Following artifact rejection, the data were segmented into 700 ms epochs from − 200 ms to + 500 ms relative to stimulus onset. Marked artifact segments were removed. Eye channels (HEOGL, VEOGI, VEOGS, HEOGR) were excluded, and baseline correction was applied using the − 200 to 0 ms pre-stimulus interval.

##### Calculation of the Target Negativity Component

After preprocessing, the calculation of the N2pc component, specifically the Target-Negativity (NT) subcomponent, was performed. For this analysis, a systematic lateralization technique (cf. Hickey et al. 2009; Woodman and Luck 2003) was employed, following the approach of Lockhofen et al. (2021). The primary difference between the groups was the selection of trials. In the TR group, only the rewarded trials suitable for NT calculations (i.e., trials in which the target stimulus was presented laterally) were selected, including both a distractor and a target-only condition. In contrast, all correct trials were selected in the NR group. As a result, the number of trials differed between the groups. However, such differences are considered negligible for this type of ERP analysis (Luck 2014).

Selected trials were averaged at each electrode to compute a single averaged waveform per condition. Electrodes relevant for the N2pc analysis were P7, PO3, P8, PO4. Ipsilateral and contralateral electrodes were defined depending on the location of the distractor or target-only stimulus. This approach accounted for the retinotopic organization of the visual system and ensured appropriate lateralization. The NT was computed by subtracting ipsilateral from contralateral ERP waveforms. A grand-average waveform across all participants was calculated to determine the NT peak for each condition, following the method outlined by Lockhofen et al. (2021). A ± 50 ms time window was defined for single-trial peak detection based on the NT peak. Peak amplitudes and latencies were extracted using the “Peak Detection” function. The NT search window was defined as 220–320 ms post-stimulus.

## Results

### Behavioral Data

**Fig. 2:**
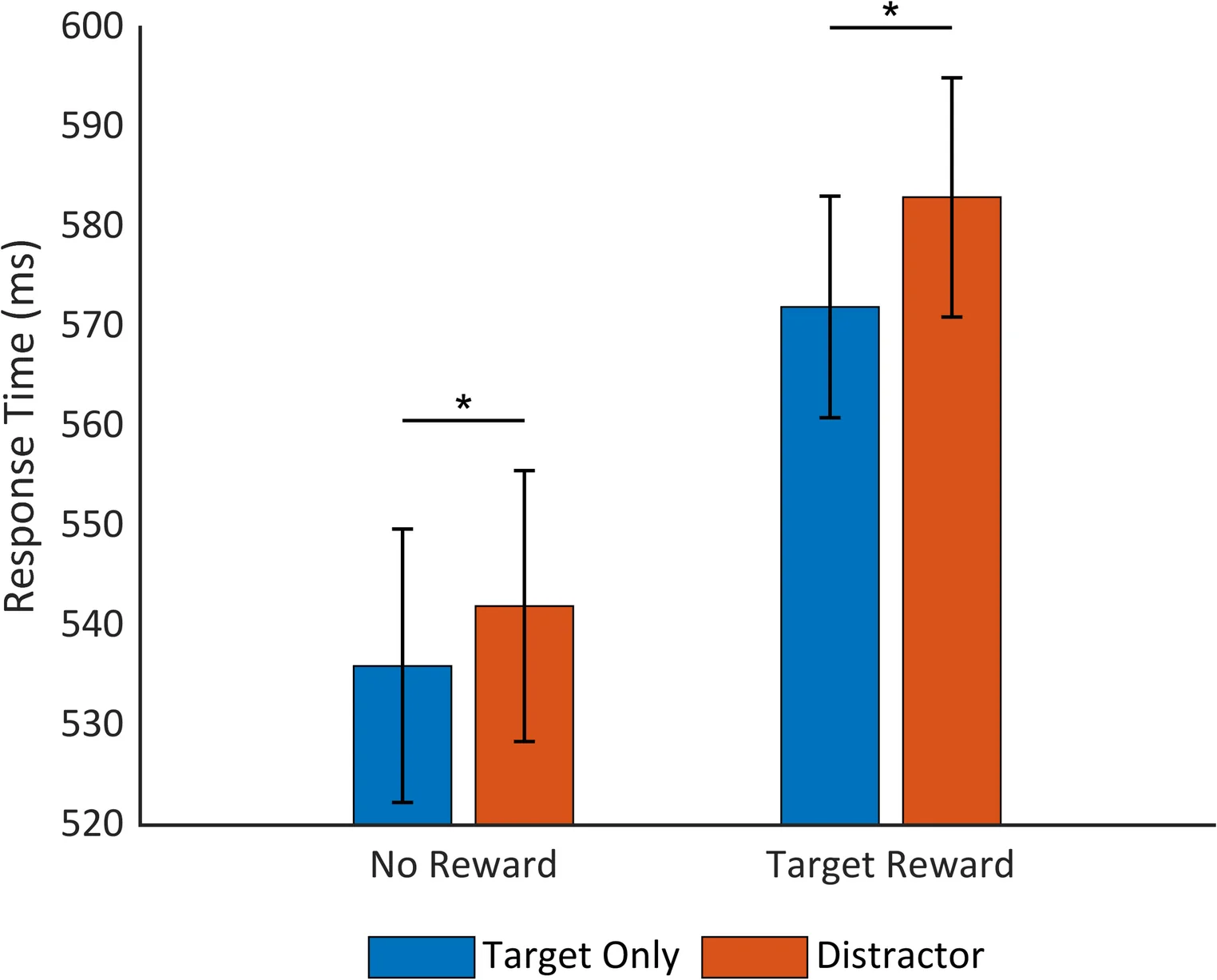
Mean response times (ms) of target only and distractor trials separated for the two reward groups (TR and NR). Blue bars represent trials with a distractor, and orange bars represent trials without a distractor. Significant differences are marked (* *p* < .05)

In the TR group, mean response times were 583 ms (SD = 50.8) for distractor trials and 572 ms (SD = 57.6) for target-only trials. In the NR group, mean response times were 542 ms (SD = 57.6) for distractor trials and 536 ms (SD = 57.9) for target-only trials. The ANCOVA with SPQ-Negative, Age, and Gender as covariates revealed a significant main effect of Group (F(1, 31) = 4.90, *p* = .034, η2p = 0.14). Participants in the TR group (*M*_*adj*_ = 581), in which the target was associated with a reward, tended to respond more slowly than participants in the NR group (M_adj_ = 537). The main effect of Distraction was also significant (F(1, 31) = 18.54, *p* < .001, η2ₚ = 0.37), indicating that response times differed between trials with and without a distractor (Fig. 2). Specifically, the presence of a distractor (*M*_*adj*_ = 563) resulted in slower response times compared to target-only trials (M = 555). The Distraction × Group interaction was not significant (F(1, 31) = 0.70, *p* = .410, η2p = 0.02), suggesting that reward association did not modulate the distraction effect. Similarly, neither the main effect of SPQ-Negative (F(1, 31) = 0.14, p =. 715, η2p = 0.004) nor the Distraction x SPQ-Negative interaction (F(1, 31) = 1.44, *p* = .239, η2p = 0.04) was significant, indicating negative schizotypal traits did not influence response times. The main effects of covariates – Age (F(1, 31) = 2.36, *p* = .135, η2p = 0.07) and Gender (F(1, 31) = 0.17, *p* = .685, η2p = 0.005) were non-significant, and their interactions with Group and Distraction did not reach significance (all *p* > .15).

### ERP-Data: NT-Component

**Fig. 3:**
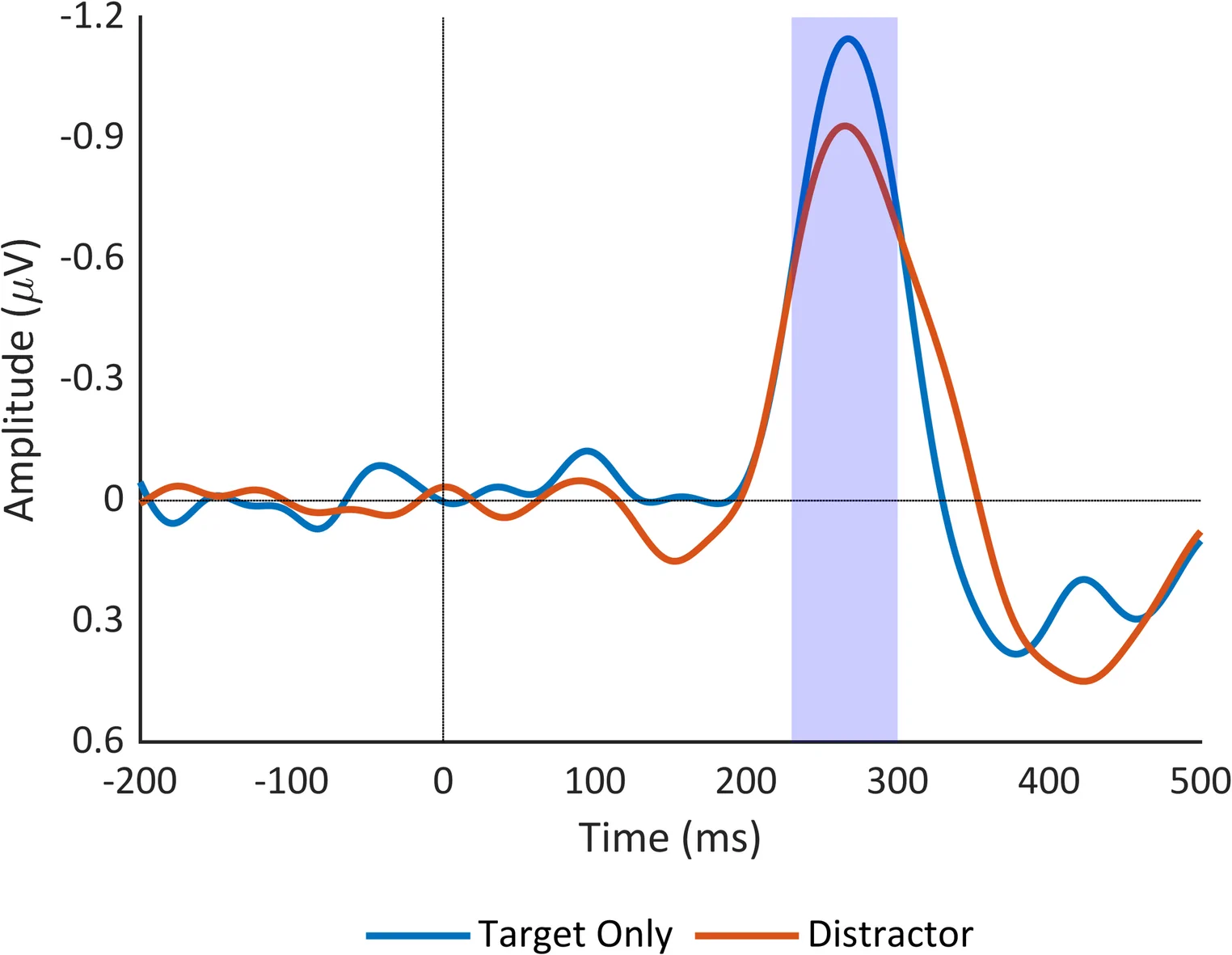
Target Negativity (NT) as a function of distraction across all groups. NT components are shown for trials with a distractor (orange) and without a distractor (blue). Significant difference is marked in blue (*p* < .05)

**Fig. 4:**
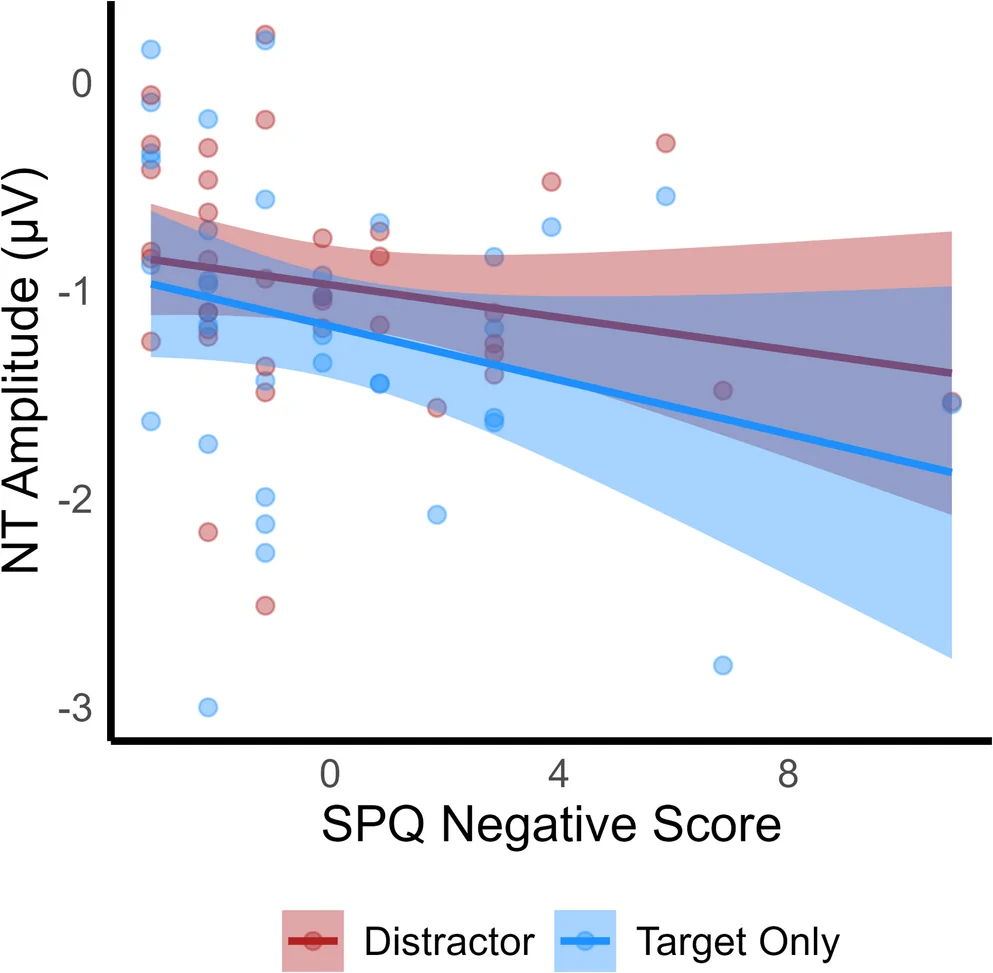
Moderation of NT amplitude by SPQ negative scores. Scatter plot illustrating the relationship between SPQ Negative scores and NT amplitudes (µV) for Target-Only (blue) and Distractor (red). Shaded areas represent 95% confidence intervals

For descriptive statistics separated by group (TR, NR), see Table 1. The ANCOVA with SPQ-Negative, Age, and Gender as covariates did not reveal a main effect of Group (F(1, 31) = 1.69, *p* = .203, ηp² = 0.05). However, there was a significant main effect of Distraction (F(1, 31) = 10.52, *p* = .003, ηp² = 0.25), with the NT being larger (more negative) in the Target-Only condition (M_adj_ = − 1.185) than in the Distractor condition (M_adj_ = − 0.993) (Fig. 3). A significant Distraction x Group interaction was also observed (F(1, 31) = 14.04, *p* < .001, ηp² = 0.31). Bonferroni-corrected pairwise comparisons indicated that NT amplitude did not differ between the distractor (Madj = -0.97) and target-only (Madj = -0.92) conditions in the TR group, t(31) = − 0.55, *p* = .587, d = − 0.20. In contrast, participants in the NR group exhibited significantly greater NT amplitude in the target-only (Madj = -1.45) condition than in the distractor condition (Madj = -1.017), t(31) = 4.97, *p* < .001, d = 1.78. In addition, a significant SPQ-Negative × Distraction interaction was observed, F(1, 31) = 8.31, *p* = .007, ηp² = 0.21. Simple slope analyses indicated that SPQ-Negative scores were significantly associated with NT amplitude in the Target-Only condition (b = − 0.096, SE = 0.033, t(31) = − 2.35, *p* = .025), but not in the Distractor condition (b = − 0.037, SE = 0.033, t(31) = − 1.12, *p* = .273). The difference between these slopes was significant (t(31) = 2.88, *p* = .007, d = 1.04), indicating that negative schizotypal traits significantly moderated the neural response differences between the target-only and distractor conditions (Fig. 4). The main effects of covariates – Age (F(1, 31) = 1.36, *p* = .252, η2p = 0.04) and Gender (F(1, 31) = 0.91, *p* = .348, η2p = 0.03) were non-significant, and their interactions with Group and Distraction did not reach significance (all ps > 0.20).

**Table 1:** Descriptive Statistics of the Target Negativity (NT) by reward group

ERP	group	mean	range	SD
NT-L [ms]	TR NR	278 276	238/319 240/319	26.5 26.8
NT-A [µV]	TR NR	-1.01 -0.94	-1.56/0.23 -2.51/-0.06	0.51 0.63
NT_TO-L [ms]	TR NR	266 279	231/301 253/312	20.8 17.2
NT_TO-A [µV]	TR NR	-1.05 -1.29	-2.80/0.20 -3.00/-0.34	0.81 0.70

Data are presented for the TR (*N* = 18) and NR (*N* = 18) groups. TR = target reward group, NR = no reward group, L = latency (ms), A = amplitude (µV), TO = target-only trials, and SD = standard deviation

## Discussion

### Summary of Results

The main aim of the present study was to investigate the behavioral and neurophysiological correlates of visual attention and reward processing and their relationship with schizotypy traits in healthy participants. Specifically, we were interested in the effects of reward and distraction on target-related processes, as indexed by the NT component of the N2pc. We found that salient, task-irrelevant distractors slowed responses compared to target only trials, irrespective of reward. On the neurophysiological level, distractors decreased target prioritization in the absence of rewards (NR group). We also found that rewarded targets increase response times compared to no reward trials. In addition, higher scores in the negative schizotypy dimension were associated with larger NT amplitudes in target-only trials, which may reflect alterations in attentional selection and top-down attentional control processes in healthy participants with higher negative schizotypy scores.

### Effects of Distraction

To assess the effects of distraction, we compared distractor trials with target only trials. Behaviorally, we found that the presence of salient distractors led to slower response times. This is in line with previous results of our group (Lockhofen et al. 2021, 2025). In visual search tasks, attention is often automatically drawn to salient or deviant stimuli, a phenomenon known as attentional capture (Theeuwes 1992,2004). At the neurophysiological level, the presence of distractors led to a reduced NT amplitude in the NR group, indicating reduced target prioritization in the absence of rewards. This result is consistent with previous findings from our group (Lockhofen et al. 2025), where distractor trials showed reduced NT amplitudes compared to target-only trials.

### Effects of Reward

In the present study, we investigated the effects of reward on target-related processes in a visual search task with salient distractors by comparing two groups: a target reward (TR) group and a no-reward (NR) group. Participants in the TR group received monetary rewards for correct responses to the target stimuli, whereas participants in the NR group received no reward.

On the behavioral level, the TR group showed a significant prolongation in response times, which is inconsistent with previous studies demonstrating that rewarding a target stimulus can decrease response times (Demeter et al. 2022; Wolf and Lappe 2023). Studies on monetary rewards in general attention tasks (Chelazzi et al. 2013; Shomstein and Johnson 2013; Yantis et al. 2012) and reward-related target stimuli (Wolf and Lappe 2023) have shown that monetary rewards lead to faster response times compared to no rewards. However, some studies indicate that factors such as the type of tasks, accuracy, and vigilance, might also influence the reward-speed relationship (Liu et al. 2020). Faster responses are typically observed in tasks requiring low vigilance (Liu et al. 2020). The present task, the AST, demands sustained attention and accuracy to receive the monetary rewards over approximately one hour. It can therefore be assumed that the AST requires a high level of vigilance over a long period of time. According to Liu et al. (2020), this may lead to slower response times, as fatigue effects could play a role in addition to the reward. Interindividual differences in reward perception may also contribute to response variability. Some participants may prioritize maximizing performance, while others may prioritize minimizing effort. This can result in variability in response times even for the same reward (Irons and Leber 2016).

On the neurophysiological level, we observed a significant Distraction x Reward group interaction. Post hoc analyses revealed that this effect was driven by a significant difference in NT amplitude between distractor and target-only trials in the NR group, whereas no such effect of distraction was found in the TR group. In the NR group, target prioritization was reduced for distractor trials compared to target only trials. This distraction effect was not present when targets were rewarded. Therefore, one can hypothesize that rewarding task-relevant targets might counteract and reduce the effect of task-irrelevant distractors. One possible explanation is that rewarding the target stimulus reduces the salience of the distractor to the extent that it no longer interferes with target processing. This is in line with another study from our group (Lockhofen et al. 2021), in which we found that participants in the TR group, compared to those in a distractor-reward group, were less susceptible to reward-related modulation.

### Effects of SPQ

Concerning the effects of schizotypy, we found an association between the negative SPQ dimension and the NT amplitude in target-only trials. Higher SPQ-Negative scores were associated with stronger NT amplitudes, suggesting enhanced target prioritization in the absence of salient distractors. However, this finding was not accompanied by faster response times. Therefore, it is possible that participants with higher SPQ-Negative scores compensate through stronger cognitive control without differences in task performance. Previous work has shown that under certain conditions, individuals with high schizotypy were able to compensate for visual processing impairments and performed normally in a working memory task (Koychev et al. 2010). These results extend the findings from a similar study conducted by our group (Lockhofen et al. 2025), which showed that ketamine administration in healthy participants increased the experience of negative symptoms and mainly affected distractor processing. The present findings suggest that negative symptoms in nonclinical individuals can also affect target processing. These results are consistent with Fuggetta et al. (2015), who reported increased target-related N2pc amplitudes in individuals with high schizotypal traits. Although there are several methodological differences between the two studies (e.g., type of search display, early vs. late phase of N2pc, absence of subcomponent analysis), the main idea that schizotypy is linked to impairments in attentional selection could also be relevant to the present findings. Attentional difficulties in individuals with high schizotypal traits have been reported in previous studies (Stotesbury et al. 2017; Moat et al. 2024; Fuggetta et al. 2015), with negative schizotypy dimension in particular being linked to attentional impairments (Alvarez-Moya et al. 2007; Chen and Faraone 2000; Smyrnis et al. 2007). This is consistent with the results of the present study, in which only the negative schizotypy dimension interacted with the distraction effect. These findings might also have implications for schizophrenia research, where cognitive deficits and perceptual disturbances are core symptoms. It has been suggested that perceptual disturbances, such as auditory hallucinations, arise from bottom-up processing abnormalities (Zhang et al. 2022), whereas cognitive deficits arise from abnormalities in top-down processing (Gold et al. 2007) or from the discrepancies between top-down predictions and sensory input (Brown and Kuperberg 2015). For example, patients with schizophrenia often experience aberrant salience, the attribution of motivational significance to otherwise irrelevant stimuli or events (Kapur 2003). Kapur (2003) proposed that hallucinations reflect the actual (bottom-up) experience of this aberrant salience, whereas delusions are the cognitive, top-down constructs that patients use to explain their experiences. This view is supported by studies showing impaired top-down regulation and enhanced bottom-up signaling in patients with schizophrenia (Lyu et al. 2025). Therefore, our finding of an association between negative schizotypy and abnormal target processing in healthy individuals are in line with the idea that impairments in top-down processing contribute to the neural mechanisms underlying schizophrenia. However, the present results must be interpreted with caution, given the small sample size and the low SPQ scores of the healthy participants included in the sample.

### Limitations

In the present study, we used the SPQ to identify associations between elevated SPQ scores and abnormalities in attentional processes. However, it should be noted that our sample consisted exclusively of healthy participants as indicated by the SPQ scores (Table 3). The mean SPQ scores of both reward groups (TR, NR) were at or below the lower cut-off threshold (12) for clinically relevant schizotypy, corresponding to approximately the 10th percentile (Raine 1991). No participants scored within the clinically elevated range (total score > 41). Consequently, caution is required when generalizing our findings to clinical populations. Future research should include participants with clinically elevated schizotypy or diagnosed schizophrenia to enable comparative analyses between clinical and non-clinical samples. Nevertheless, the present study provides valuable insights that may inform future investigations. Additionally, the present study focused exclusively on the N2pc component and analyzed only parieto-occipital electrodes (P7, PO3, P8, PO4). Previous research has indicated that other regions, such as frontal areas, may also play a role in reward-modulated attention (Lockhofen et al. 2021). This may explain why, despite observing behavioral differences in response times related to reward, no corresponding neurophysiological effects were detected. Future research should therefore include additional brain regions through complementary approaches such as source-space analyses.

### Conclusion

The present study demonstrates that target processing can be impaired by salient but task-irrelevant distractors. This effect of distraction on target prioritization was more pronounced in the NR group which might indicate that rewarding targets can reduce the effect of distractors. Furthermore, higher scores in the negative schizotypy dimension were associated with enhanced target prioritization, particularly in the absence of distractors. These findings are consistent with previous studies reporting abnormalities in visual attention and cognitive control processes in patients with schizophrenia, healthy subjects under ketamine, and those with high schizotypy. The present findings extend this evidence by showing that not only salience-driven processes but also target-related processes are modulated by reward, distractors, and possibly individual differences in negative schizotypy.

## Supplementary Information

Below is the link to the electronic supplementary material.

Supplementary Material 1

## Data Availability

No datasets were generated or analysed during the current study.
