# Supplementary material for: Neurophysiological Correlates of Target-Related Visual Attention Processes as a Function of Reward in Connection with Schizotypy
Source: Brain Topogr. 2026 Oct 10;39(6):102. doi: 10.1007/s10548-026-01260-0 (PMC13653958; doi:10.1007/s10548-026-01260-0)
Supplement: Supplementary file 1 [file 10548_2026_1260_MOESM1_ESM.docx]

**Statistical Analysis**

Behavioral and ERP data were analyzed using mixed-design ANCOVAs with Distraction (Distractor, Target-Only) as a within-subjects factor, Group (TR, NR) as a between-subjects factor, and mean-centered Age, Gender, and SPQ scores as covariates. Separate models were run for the SPQ Total score and its Disorganized, Positive, and Negative subscales. Only the SPQ Negative subscale showed a significant interaction in ERP and was reported in the manuscript. However, results for the other models were computed and reported in the supplementary material for completeness.

**Results**

**Behavioral Data**

First, using the SPQ Total score as a covariate, the ANCOVA revealed a significant main effect of Group, *F*(1,31) = 4.53, *p* = .041, *η_p_^2^* = .13, with the TR group (*M_adj_* = 578) responding more slowly than the NR group (*M_adj_* = 539). A significant main effect of Distraction was also observed, *F*(1, 31) = 19.62, *p* < .001, *η_p_^2^* = .39, as the presence of a distractor (*M_adj_* = 563) resulted in slower response times than target-only trials (*M_adj_* = 555). Additionally, all covariates – SPQ-Total, *F*(1, 31) = .13, *p* = .723, *η^2^_p_* = .004, Age, *F*(1, 31) = 2.26, *p* = .143, *η^2^_p_* = .07, and Gender, *F*(1, 31) = .07, *p* = .800, *η^2^_p_* = .002, were non-significant. No significant interactions involving Group and Distraction were found (p >.05).

Regarding the SPQ Disorganized subscale, the ANCOVA also revealed a significant main effect of Group, *F*(1,31) = 4.95, *p* = .033, *η_p_^2^* = .14, with the TR group (M_adj_ = 579) responding more slowly than the NR group (M_adj_ = 539). A significant main effect of Distraction was also observed, *F*(1,31) = 1 8.22, *p* < .001, *η_p_^2^* = .37, as the presence of a distractor (M_adj_ = 562) increased response times compared to target-only trials (M_adj_ = 555). As with the total subscale, all covariates – SPQ-Total, Age, and Gender were non-significant (all p > 13). No significant interactions involving Group and Distraction were found (p >.14).

Regarding the SPQ Positive subscale, the ANCOVA revealed a significant main effect of Group, *F*(1,31) = 4.87, *p* = .035, *η_p_^2^* = .14, with the TR group (M_adj_ = 579) responding more slowly than the NR group (M_adj_ = 539). A significant main effect of Distraction was also observed, *F*(1,31) = 20.43, *p* < .001, *η_p_^2^* = .40, in which the presence of a distractor (M_adj_ = 563) increased response times compared to target-only trials (M_adj_ = 555). While the Group × Distraction interaction was not significant, a significant interaction was found between SPQ-Positive and Distraction, *F*(1,31) = 5.26, *p* = .029, *η_p_^2^* = .15. Simple slopes analysis revealed that while the relationship between SPQ-positive scores and response time was not significant within the Distractor condition (t(31) = 0.76, p = .450) or the Target-Only condition (t(31) = 0.35, p = .728) individually, the difference in response times between these two conditions significantly changed as SPQ-Positive scores increased, *t*(31) = 2.29, *p* = .029, *d* = .82. This suggests that higher levels of SPQ-Positive symptoms moderate the impact of distraction on reaction time. Covariates for Age and Gender, as well as their interactions with Distraction, did not reach significance (all *p* > .09).

**ERP Results**

Using SPQ total score as a covariate, the ANCOVA revealed a significant main effect of Distraction, F(1, 31) = 8.71, p = .006, ηp² = .22. This effect was qualified by a significant Group × Distraction interaction, F(1, 31) = 6.88, p = .013, ηp² = .18. Bonferroni-adjusted pairwise comparisons indicated that NT amplitude did not differ between the distractor (*M_adj_* = -1.00) and target-only ((*M_adj_* = -1.02) conditions in the TR group, t(31) = 0.21, p = .839, d = 0.07. In contrast, participants in the NR group exhibited significantly greater NT amplitude in the target-only (*M_adj_* = -1.35) condition than in the distractor (*M_adj_* = -0.98) condition, t(31) = 3.95, p < .001, d = 1.42. No significant main effects of Group, SPQ total score, age, or gender, nor any other interactions, were observed (all ps > .18).

Using the SPQ-Disorganized subscale as a covariate, the ANCOVA revealed a significant main effect of Distraction, F(1, 31) = 8.70, p = .006, ηp² = .22. This effect was qualified by a significant Group × Distraction interaction, F(1, 31) = 6.32, p = .017, ηp² = .17. Bonferroni-adjusted pairwise comparisons indicated that NT amplitude did not differ between the distractor (*M_adj_* = -1.02) and target-only (*M_adj_* = -1.05) conditions in the TR group, t(31) = 0.33, p = .747, d = 0.12. In contrast, participants in the NR group exhibited significantly greater NT amplitude in the target-only (*M_adj_* = -1.34) condition than in the distractor (*M_adj_* = -0.97) condition, t(31) = 3.86, p < .001, d = 1.39. No significant main effects of Group, SPQ-Disorganized score, age, or gender, nor any other interactions, were observed (all ps > .18).

Using the SPQ-positive subscale score as a covariate, the ANCOVA revealed a significant main effect of Distraction, F(1, 31) = 8.87, p = .006, ηp² = .22. However, this effect was qualified by a significant Group × Distraction interaction, F(1, 31) = 6.10, p = .019, ηp² = .16. Bonferroni-adjusted pairwise comparisons indicated that NT amplitude did not differ between the distractor (*M_adj_* = -1.01) and target-only (*M_adj_* = -1.05) conditions in the TR group, t(31) = 0.37, p = .712, d = 0.13. In contrast, participants in the NR group exhibited significantly greater NT amplitude in the target-only (*M_adj_* = -1.34) condition than in the distractor (*M_adj_* = -0.98) condition, t(31) = 3.85, p < .001, d = 1.38. No significant main effects of Group, SPQ-positive score, age, or gender, nor any other interactions, were observed (all ps > .17).
